# Genomic, Transcriptomic and Epigenomic Tools to Study the Domestication of Plants and Animals: A Field Guide for Beginners

**DOI:** 10.3389/fgene.2020.00742

**Published:** 2020-07-15

**Authors:** Josué Barrera-Redondo, Daniel Piñero, Luis E. Eguiarte

**Affiliations:** Departamento de Ecología Evolutiva, Instituto de Ecología, Universidad Nacional Autónoma de México, Mexico City, Mexico

**Keywords:** population genomics, pangenomics, ancient DNA, differential expression analysis, epialleles, genome editing

## Abstract

In the last decade, genomics and the related fields of transcriptomics and epigenomics have revolutionized the study of the domestication process in plants and animals, leading to new discoveries and new unresolved questions. Given that some domesticated taxa have been more studied than others, the extent of genomic data can range from vast to nonexistent, depending on the domesticated taxon of interest. This review is meant as a rough guide for students and academics that want to start a domestication research project using modern genomic tools, as well as for researchers already conducting domestication studies that are interested in following a genomic approach and looking for alternate strategies (cheaper or more efficient) and future directions. We summarize the theoretical and technical background needed to carry out domestication genomics, starting from the acquisition of a reference genome and genome assembly, to the sampling design for population genomics, paleogenomics, transcriptomics, epigenomics and experimental validation of domestication-related genes. We also describe some examples of the aforementioned approaches and the relevant discoveries they made to understand the domestication of the studied taxa.

## Introduction

The modern study of domestication of plants and animals is multidisciplinary, and relevant contributions come from botany, zoology, archeology, genetics, ethnobiology, biogeography, and linguistics (Larson et al., 2014). Modern domestication studies seek to understand the dates of domestication, the places where domestication started and number of times that domestication took place, as well as the details of the evolutionary and ecological forces that led to the divergence between the domesticated taxa and their wild relatives and ancestors (Zeder, 2006; Larson et al., 2014).

Given that domestication is an evolutionary process, genetics emerged as a powerful tool to understand the domestication of plants and animals, revealing the demographic history of the domesticated taxa and the genetic variants that underlie their domesticated phenotypes (Zeder et al., 2006; Gepts, 2014). The advent of high-throughput sequencing technologies sparked the use of genomic studies to understand the domestication of crops and animals in a much deeper level than previously imagined, as researchers can now pinpoint the genetic changes that allowed domestication to happen (Ross-Ibarra et al., 2007; Gepts, 2014).

## Why and How to Use a Genomic Approach in Domestication Studies? Top-Down and Bottom-Up Approaches for the Study of Domestication

In genetics, we refer to *top* or *up* when referring to a specific phenotype, while we refer to *bottom* or *down* when referring to the underlying genotype responsible for that trait. Thus, top-down approaches start by studying a particular phenotype and searching for its genetic basis. Huge advances in the genetic study of domestication traits have been made using classic top-down approaches (*e.g*., Sax, 1923; Paterson et al., 1988; Doebley and Stec, 1991; Doebley et al., 1995), which are performed by analyzing the phenotypic traits of interest between wild and domesticated taxa, and then finding the genetic variant or variants that correlate with the phenotypic traits through the mapping of quantitative trait loci and linkage disequilibrium (Ross-Ibarra et al., 2007; Kantar et al., 2017). These top-down approaches are precise in finding causal variants involved in the evolution of specific traits, but usually they are very labor-intensive and are biased towards *a priori* selected phenotypes to be compared between wild and domesticated taxa (Ross-Ibarra et al., 2007; Kantar et al., 2017).

In contrast to top-down approaches, bottom-up approaches start by analyzing the genetic variation within genomes in order to detect potential signals of selection related to the domestication process and finally associate such evolutionary signals to important loci and domestication phenotypes (Ross-Ibarra et al., 2007; Kantar et al., 2017). In the last decade, high-throughput sequencing technologies allowed us to analyze entire genomes of one or several individuals of domesticated taxa, and to compare them to different varieties or to their wild relatives (*e.g.*, Hufford et al., 2012; Yang et al., 2012; Li et al., 2013; Wang et al., 2019; Zeng et al., 2019).

Bottom-up approaches do not need an *a priori* phenotypic target, enabling a genome-wide search of domestication-related loci without previous background of possible candidates, revealing important traits that can hardly be studied using a top-down approach (Ross-Ibarra et al., 2007; Kantar et al., 2017). Nevertheless, the results of bottom-up approaches can be limited by the sampling scheme, the density of genetic markers, and the detection of false positives (Tiffin and Ross-Ibarra, 2014), so these genomic approaches have to be properly and carefully designed in order to obtain satisfying results (De Mita et al., 2013).

Genomic data facilitated the widespread and reliable use of bottom-up approaches to study plant and animal domestication, but top-down strategies were also aided by genomics, allowing a more efficient search of genotype-phenotype correlations through genome-wide association studies (GWAS; Wang G.-D. et al., 2014), which can be defined as experimental designs that are used to detect the association between genetic variation in a population and phenotypical traits of interest (Visscher et al., 2017).

Genome-wide genetic markers allows to differentiate between global and local evolutionary signals occurring throughout the genome (Diao and Chen, 2012), discerning the signals of selection during domestication (Vitti et al., 2013) from other fine-scale signals of demographic events that occurred during the domestication process (Meyer and Purugganan, 2013; Guerra García and Piñero, 2017).

The use of modern genomic tools is not limited to population genetics, as other interesting approaches can reveal important aspects of the domestication process. For instance, one can analyze changes in the transcriptional activity of genes related to domestication (Hekman et al., 2015), demonstrate the phenotypic effects of certain alleles through the use of genomic editing tools (Zhou J. et al., 2019), search for epigenetic patterns that changed between domesticated and wild taxa (Janowitz Koch et al., 2016) or analyze the genetic makeup of archeological samples (Irving-Pease et al., 2019).

**FIGURE 1:**
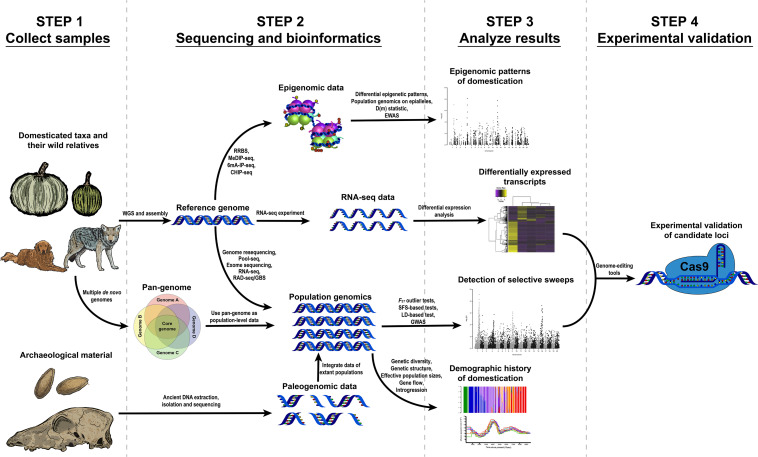
Proposed workflows to study different problems related to the domestication of plants and animals through genomic, transcriptomic and epigenomic tools.

This review describes the necessary steps and data to start a genomic research project towards understanding domestication, the questions that can be approached using genomic data and the main results obtained from previous studies using these methods (Figure 1).

## Whole-Genome Assembly and Reference Genomes

Whole-genome assembly is one of the first steps in modern domestication studies, since it generates a reference genome that is useful for downstream analyses. Whole-genome assembly projects require the use of high-throughput sequencing technologies such as Illumina (*e.g.*, Sun et al., 2017), PacBio (*e.g.*, Badouin et al., 2017; VanBuren et al., 2018), Oxford Nanopore (*e.g.*, Belser et al., 2018) or a combination of these (*e.g.*, Bickhart et al., 2017; Zhou Y. et al., 2019) to sequence the genome of interest of a single individual. Before starting a genome assembly project, a rough estimate of the haploid genome size must be known as well as the ploidy of the organism, since the assembly difficulty and sequencing cost are determined by both factors (Sims et al., 2014). In order to successfully assemble eukaryotic genomes, where repetitive elements usually comprise a significant portion of its content [ranging from 3% in tiny genomes such as *Utricularia gibba* (Ibarra-Laclette et al., 2013) up to 65.5% in huge genomes such as *Ambystoma mexicanum* (Nowoshilow et al., 2018)], it is necessary to generate sequencing libraries with large insert sizes – called mate-pair libraries – or use long-read sequencing technologies such as PacBio or Oxford Nanopore (Levy and Myers, 2016; Sohn and Nam, 2016). Additionally, the use of chromosome conformation capture (Mascher et al., 2017), optical mapping (Dong et al., 2013) or linkage maps obtained from crosses (Fierst, 2015) will help achieve chromosome-level assemblies that are highly desirable to adequately assess haplotypes, linkage disequilibrium, putative genomic rearrangements and the genomic location of candidate loci (Sohn and Nam, 2016; see Table 1).

**TABLE 1:** List of key publications with other reviews that are focused on specific topics, as well as some notable examples of research articles using some of the methods described in this review with reliable results.

**Topic**	**Citation**	**Usefulness/importance**
Genome assembly and reference genomes	Sohn and Nam, 2016	In-depth review on genome assembly. Includes compelling explanations behind the genome assembly algorithms and an extensive list of genome assembly strategies.
	Yandell and Ence, 2012	In-depth review on eukaryotic genome annotation, a description of the available tools to predict genes and best practices when predicting genes.
Sequencing strategies	Meirmans, 2015	Classic review concerning common pitfalls that should be avoided in a population genomic study. A compulsory review for any newcomer to population genomics.
	Dorant et al., 2019	Empirical study that compares the efficiency of Pool-seq, RADseq and Rapture to detect weak signals of genetic structure in lobsters.
	Inbar et al., 2020	Empirical study that compares the efficiency of whole-genome sequencing, Pool-seq and RADseq for GWAS in ants.
Pan-genomics	Golicz et al., 2016a	In-depth review about pan-genomics in plant species, its advantages over the use of reference genomes, a guide on how to generate pan-genomes and the importance of studying structural variants. The article is dedicated to plants, but the rationale and methods can also be applied to other eukaryotes.
	Khan et al., 2020	Opinion article detailing the relevance of pan-genomes as a necessary next step from reference genomes. The authors also highlight the importance of including wild taxa into pan-genomics and propose the idea of genus-level super-pan-genomes.
	Gao et al., 2019	Landmark study of the tomato pan-genome. The authors sequenced 725 accessions from the domesticated tomato and its wild relatives. They found 4,873 additional genes, including several well-characterized genes that were absent from the reference-genome. They also evaluated the presence-absence variants between the wild and domesticated tomatoes, which were enriched in disease-resistance genes.
Demographic analyses	Linck and Battey, 2019	Research study focused on the effects of minor allele-frequency filters to detect genetic structure in populations. Gives a good explanation on the rationale behind the clustering-based methods to detect structure.
	Mather et al., 2020	Review dedicated to the theoretical background and technical requirements of PSMS and MSMC to infer changes in effective population sizes and coalescent times.
	Gerbault et al., 2014	Excellent review on how to use Bayesian approaches to test different demographic models of domestication.
	Frantz et al., 2015	Landmark study on pig domestication. The authors make use of Approximate Bayesian computation to compare domestication scenarios, they use clustering-based methods to detect genetic structure and used a graph-based method to infer the genetic relationship between pig and wild boar populations.
Selection scans	Vitti et al., 2013	Good review focused on explaining the rationale behind many of the bottom-up tests to detect selection and the genomic signals they are sensitive to.
	De Mita et al., 2013; Lotterhos and Whitlock, 2015	Classic simulation-based studies that compare different scenarios to evaluate the best sampling strategies and the most powerful methods to detect selection throughout the genome, according to the reproductive nature of the organism under study.
	Gibson, 2018	A primer dedicated to understanding the principles behind GWAS and its ability to detect polygenic effects on quantitative traits.
	Hufford et al., 2012	A landmark paper that illustrates how to perform genome scans to detect domestication-related loci in domesticated taxa, and the importance of these loci for crop improvement. The paper studies the domestication of maize, but a similar study design can be applied to domesticated animals.
Paleogenomics	Irving-Pease et al., 2019	Exhaustive book chapter dedicated to the study of ancient DNA to understand domestication.
	Allaby et al., 2019	Research study that casts into doubt the long-lasting idea that domestication processes lead to strong population bottlenecks by re-analyzing data based on ancient DNA samples.
	Daly et al., 2018	Remarkable study that sequenced and analyzed 83 mitochondrial genomes and 51 nuclear genomes from ancient goat samples. The authors found signals of ancient introgression events, as well as ancient selective signals related to several traits that are shared with modern goats.
Transcriptomics	Fang and Cui, 2011	General guideline on how to adequately design an RNA-seq experiment to avoid technical mistakes and generate meaningful results.
	Yang and Kim, 2015	General guideline on how to analyze RNA-seq data to assess differential expression.
	Hekman et al., 2015	In-depth review dedicated to study the domestication process through transcriptomics, including methodological strategies and challenges.
	Hradilová et al., 2017	An excellent study that combines transcriptomic data with metabolomic data and morphological data between domesticated and wild peas. The analysis of multi-omic data allowed them to get a better understanding behind seed dormancy and pod dehiscence in domesticated peas.
Epigenomics	Guerrero-Bosagna, 2012; Heard and Martienssen, 2014; Burggren, 2016	Contrasting views on the role of transgenerational epigenetic inheritance in evolution. The topic is still debated and should be viewed critically.
	Jensen, 2015	In-depth review on the rationale and advances of epigenetic studies to understand domestication. The manuscript is focused on animal behavior, but many of the ideas can also be applied to domesticated plants.
	Janowitz Koch et al., 2016	A landmark paper showing the importance of epigenetic marks on dog domestication and its association with behavioral traits. The study doesn’t just compare the methylation marks between wolves and dogs, but also assess the heritability of the methylation marks and proposes a formal test to detect selection on epialleles.
Genome-editing tools	Boettcher and McManus, 2015).	Review on novel genome-editing techniques and RNA interference. Useful to compare and choose the best tool to validate candidate loci.
	Shan et al., 2020	A general guide on how to develop a CRISPR/Cas9 system on a non-model plant species.
	Soyk et al., 2017	Landmark paper that uses genome-editing to validate two candidate genes related to fruit size and reduced fruit dropping in tomato. The authors also detect the emergence of undesirable traits in domesticated tomatoes due to an epistatic effect between both domesticated loci and introduce wild alleles to generate new tomato phenotypes with reduced degrees of the undesirable traits.
Perspectives	Piperno, 2017	Review centered on the potential application of an extended synthesis framework to understand domestication. Centered around the concepts of niche construction, transgenerational epigenetic inheritance and developmental plasticity.

After sequencing and assembling the genome of at least one individual, it must be properly annotated before it can be of any use. Since eukaryotic genes are structurally complex, genome assemblies require the additional sequencing of RNA data from the same species to be used as transcriptomic evidence, alongside homology evidence from other curated genomes and *ab initio* predictions based on the underlying structure of genes, in order to be successfully annotated (Yandell and Ence, 2012; see Table 1). Even though whole-genome assembly projects were previously restricted to large research groups (*e.g.*, Schnable et al., 2009; Tomato Genome Consortium, 2012), the sequencing cost per nucleotide is declining constantly in all the aforementioned technologies, making genome analyses accessible for a large part of the research community (Muir et al., 2016). The current bottleneck for small research groups is usually not the cost of sequencing itself, but rather the availability of computational resources capable of storing and analyzing huge amounts of data (Muir et al., 2016).

The main purpose of assembling a genome in a domestication study is to use it as a reference for high-quality population data to infer the selection, introgression and recombination processes, and to design posterior studies for experimental validation of candidate loci. Even though several population-level analyses based on reduced-representation genome sequencing can be performed in the absence of a reference genome (De Wit et al., 2012; Mastretta-Yanes et al., 2015), the use of a reference genome alongside population data enables the correct identification of otherwise anonymous loci into specific genes or regions within the genome and it makes possible the identification and the proper handling of linkage between loci (Fitz-Gibbon et al., 2017). Also, it can help to discriminate between orthologous and paralogous loci, which is critical given the large size of many genomes and the frequent genome duplication processes experienced during the evolution of plant and animal lineages (Clark and Donoghue, 2018; Zadesenets and Rubtsov, 2018).

Thus, the availability of a reference genome is desired for genomic analyses concerning domestication. Luckily, domesticated taxa are usually economically relevant, drawing the attention of several research groups worldwide and in some cases helping to fund the projects. Therefore, reference genomes are usually available for domesticated species, since such data is also relevant for other research areas, such as crop improvement and breeding programs (Ellegren, 2014). However, it should be noted that using a single reference genome can lead to reference bias, where sequenced individuals that are more distantly related to the reference will tend to have fewer predicted variants due to mismatches while mapping the reads (Günther and Nettelblad, 2019).

Besides its use as a reference genome for population-level data, the analysis of several whole-genome assemblies between domesticated and wild taxa will help us reveal structural differences between the genome of a domesticated taxon and its closest wild relatives, such as duplications, chromosome rearrangements or presence/absence of entire genes and genomic regions (Yang et al., 2012; Wang W. et al., 2014; Xie et al., 2019). Since selection and bottlenecks during domestication often leads to the fixation of mutations that involve a loss of function (Renaut and Rieseberg, 2015; Moyers et al., 2018), comparative analyses using genome assemblies of wild ancestors may also reveal these changes in genes that could not be properly predicted within the domesticated genome (Moyers et al., 2018). In this sense, further efforts should be made to assemble high-quality genomes of wild relatives alongside the domesticated taxon of interest (Brozynska et al., 2016; Xie et al., 2019).

## Strategies to Gather Adequate Population Genomics Data

Genome assemblies alone give us a limited view on domestication, unless several genomes of wild relatives (if known and available) and domesticated individuals are sequenced, because evolution is a population-level process, and in consequence population data is necessary to address most of the evolutionary questions in domestication (Wang G.-D. et al., 2014; Guerra García and Piñero, 2017). Population genomics examines the genetic variation within and between populations that is scattered across the entire genome to assess the demographic history, phylogenetic relations and selective pressures of a species (Jorde, 2001). Several types of genomic data can be evaluated at the population level, including single nucleotide polymorphisms (SNPs), indels and copy number variations; but SNPs are the most commonly analyzed of the three (Seal et al., 2014).

All population-level sequencing techniques share common pitfalls that should be known and avoided before investing any money on sequencing. Population sampling should be planned carefully, as the sampling scheme has a stronger impact over sequencing to obtain reliable results in any analysis (Meirmans, 2015). Also, different populations should be mixed, rather than being sequenced on separate libraries or sequencing lanes, as failing to do so will generate sequencing biases that can be confused with biological patterns (Meirmans, 2015; see Table 1).

Once adequate genomic population data is gathered, we need to analyze the demographic processes that shaped the genetic variation and the population structure of contemporary populations during the domestication process. This data is necessary to perform tests to detect natural and artificial selection, which are required to understand the genetic base of domestication syndromes (Ross-Ibarra et al., 2007). There are several approaches to obtain population data at a genomic scale, which differ in the fraction of the genome that is sequenced, therefore determining the sequencing cost of each sample (Schreiber et al., 2018).

### Whole-Genome Sequencing of Populations

After assembling a reference genome, one of the next possible strategies to understand domestication is to sequence the complete genome of several individuals. This approach requires the alignment of the sequencing reads back to a reference genome, in order to infer the variable sites between individuals and know the genetic elements (e.g., genes, upstream regulators, repetitive elements, non-coding RNAs) associated to those sites. The main benefit of this approach is its potential to retrieve all the variant sites within an individual’s genome that are structurally represented in the reference genome. Whole-genome sequencing can be used in almost any population-level test of interest (Schreiber et al., 2018). Common practices recommend a sequencing depth around 30× per individual, but empirical studies in pigs suggests that even 10x is enough to cover up to 99% of a genome with accurate detection of variant sites (Jiang L.G. et al., 2019). The main drawback of this approach is the sequencing cost of each sample, which is significantly higher compared with other approaches, especially for organisms with large genomes such as polyploid crops or mammals (Schreiber et al., 2018). This can lead researchers to evaluate a trade-off between sequencing depth and number of sampled individuals to optimize their resources. Simulation studies suggest that sequencing more individuals is more convenient to obtain reliable results, even at the expense of lower sequencing depths per individual (Fumagalli, 2013).

### Alternatives to Whole-Genome Sequencing

Other approaches aim to reduce the sequencing cost per samples by pooling the DNA of several individuals into a single sequencing library (Futschik and Schlötterer, 2010) or by reducing the portion of the genome that is sequenced (often named as reduced-representation sequencing), either by sequencing arbitrary defined segments scattered across the genome, by targeting the desired portions of the genome or by sequencing the transcriptionally active portions of the genome (Schreiber et al., 2018). These techniques are especially helpful for organisms with very large genomes, and some of these methods can even be used in the absence of a reference genome (Mastretta-Yanes et al., 2015; Schreiber et al., 2018). Furthermore, the reduced representation of the genome means that those fewer regions that are targeted can have a high sequencing depth, leading to higher accuracy of the observed genetic variation and better heterozygosity estimations (Schreiber et al., 2018). Additionally, the reduced sequencing cost per sample allows for a large number of sequenced individuals and populations that, with a proper sampling strategy, can lead to robust results (De Mita et al., 2013; Lotterhos and Whitlock, 2015). Due to the fragmented nature of these sequencing techniques, reduced representation data alone may be insufficient to pinpoint all or even the most important possible causal genetic variants associated to the domestication syndromes (Lowry et al., 2017), but they are still useful to infer basic genetic statistics, infer demographic properties and past demographic scenarios, detect some signatures of selective sweeps across the genome and even perform GWAS for domestication traits of interest (Andrews et al., 2016; Schreiber et al., 2018).

### Pool Sequencing

Pool sequencing (Pool-seq) is a promising alternative to whole-genome sequencing with a much lower cost (Futschik and Schlötterer, 2010). As the name suggests, Pool-seq consists of sequencing a large pool of individuals for a given population into a single high-throughput sequencing library, instead of sequencing each individual separately, allowing an accurate estimation of allele frequencies and other parameters of population genetics at the expense of losing individual-level information (Futschik and Schlötterer, 2010). This method requires to map the reads against a reference genome of the same species in order to work (Schlötterer et al., 2014). It is intended for sequencing large pools of individuals (>40 individuals per population is recommended, but >100 is optimal), otherwise the allele frequencies will not be estimated accurately (Schlötterer et al., 2014). The relative amount of pooled DNA of each individual in a Pool-seq study should be similar in order to avoid overrepresentation of individual alleles, a task that is often challenging (Schlötterer et al., 2014).

Pool-seq has several limitations that should be considered based on the objectives of the research project. It is difficult to discard a low-frequency allele from a sequencing error, but this problem is potentially fixed by either establishing a minor allele frequency threshold for SNP calling or by using pool replicates (Schlötterer et al., 2014). One important limitation is the inability of Pool-seq data to estimate linkage disequilibrium and haplotype phasing, which is particularly important to evaluate the non-independence of genetic signals in demographic studies and selective scans (Schlötterer et al., 2014). Finally, assessing genetic structure can be difficult and sometimes misleading when using Pool-seq, due to potential biases in individual allele representations within the pool (Dorant et al., 2019). This makes Pool-seq an adequate method for GWAS, selective sweeps and some methods based on allele frequencies when resources are limited (Luu et al., 2017; Inbar et al., 2020), but the loss of individual-level information makes many of the demographic inferences difficult, as populations need to be predefined before sequencing (Dorant et al., 2019), and the bioinformatic tools that handle Pool-seq data are scarce.

### Exome Capture and Sequencing

Exome sequencing is another lower-cost alternative to whole-genome sequencing which targets the protein-coding regions of the genome (Warr et al., 2015; Kaur and Gaikwad, 2017). Protein-coding genes represent a small fraction of eukaryotic genomes, which is particularly useful for most population genomic studies, since it represents mostly functional elements within genomes (Kaur and Gaikwad, 2017). This technique is usually performed using hybridization probes, which requires previous knowledge of the genome content as well as a *priori* selection of regions of interest in order to design probes (Kaur and Gaikwad, 2017). Fortunately, hybridization probes are already available for several domesticated plants and animals (Warr et al., 2015; Kaur and Gaikwad, 2017).

Despite its advantages, exome sequencing can generate an uneven sequencing depth in certain genomic positions, unlike whole-genome sequencing that shows a uniform distribution of reads throughout the genome (Lelieveld et al., 2015). Another important limitation of exome sequencing is its bias towards the protein-coding portion of the genome, since increasing evidence shows that many of the genetic changes that have been directly associated to domestication traits are located within *cis*-regulatory elements, noncoding RNAs and other *trans*-regulatory elements, rather than within the open reading frame of the genes (Swinnen et al., 2016). Despite its limitations, demographic history and selective sweeps can still be detected using this sequencing method (Pankin et al., 2018).

### RNA Sequencing of Populations

Transcriptome sequencing (also known as RNA-seq) is another useful approach to obtain population-level data from the transcriptionally active elements within genomes (De Wit et al., 2012). RNA-seq can be mapped against a reference genome to detect genetic variants and determine the genomic regions of interest, but it can also be analyzed in the absence of a reference genome (De Wit et al., 2012), since transcriptomes can be assembled *de novo* (Haas et al., 2013) and the functional annotation of the assembled transcripts is relatively easy (Bryant et al., 2017).

However, transcription profiles are dependent on the sequenced tissues and organs, the development stage of the organism, and the influence of external stimuli, capturing just the transcripts that are active at the moment of RNA extraction (Hekman et al., 2015). This complexity can generate important biases in the relative abundance of certain transcripts over others and overlook potential adaptative genes whose expression are context dependent (Hekman et al., 2015; Kaur and Gaikwad, 2017). Nonetheless, RNA-seq is still a good option for species with large genomes that are hard to assemble (De Wit et al., 2012). Similarly to exome-sequencing, RNA-seq data can be used to evaluate demographic history and selective sweeps, but the selective signals are restricted to the transcriptionally active part of the genome, and cannot be used to evaluate structural variants (Schreiber et al., 2018).

### Restriction Site-Associated DNA Sequencing

Restriction site-associated DNA sequencing (RAD-seq), which may also be referred to as genotyping by sequencing (GBS), has been one of the most popular options for cost-affordable population genomics in the last decade (Davey and Blaxter, 2010). The technique consists in using restriction enzymes to digest the DNA and sequence the regions adjacent to the restriction sites that are scattered across the genome (Davey and Blaxter, 2010). It can also be combined with sequence capture techniques to target specific loci of interest (Ali et al., 2016). RADseq data can either be mapped against a reference genome or it can be assembled *de novo* (Catchen et al., 2013; Mastretta-Yanes et al., 2015), making it a versatile technique for species with scant genomic resources.

However, empirical studies show that using certain *de novo* approaches for RAD-seq data can lead to fewer predicted SNPs due to errors in the definition of loci and treatment of sequencing errors (Shafer et al., 2017), all which may subsequently alter downstream analyses, especially those based on the distribution of allele frequencies within the genome of a population, also known as the site frequency spectrum (SFS) (Shafer et al., 2017). For this reason, a reference-based approach is highly recommended as long as the reference genome is closely related to the population dataset (Shafer et al., 2017). Furthermore, RADseq data could involve errors when a polymorphism resides within a restriction site, which prevents the enzyme to cut in individuals carrying such polymorphism, leading to failures in sequencing that region in homozygous individuals (null alleles) and makes heterozygous individuals to look like homozygotes (allele dropout) (Andrews et al., 2016). Finally, the capacity of RADseq libraries to adequately perform selective scans has been casted into serious question (Lowry et al., 2017). Its potential capacity to detect selective sweeps is dependent on the genome size, the density of variants detected for a given genomic region and specially the length of the extent of linkage disequilibrium in the genome (Lowry et al., 2017). Thus, when a species genome has short regions in linkage disequilibrium (due to high recombination rates) and the SNP density is low (particularly in large genomes), odds are that the selective scans will likely miss a significant portion of selective sweeps associated to domestication (Lowry et al., 2017).

## Pan-Genome Analyses in Domesticated and Wild Taxa

An increasing number of studies are revealing that structural variants (copy-number variation, presence/absence of genomic regions, inversions, transversions, translocations) are common within plant and animal populations (Khan et al., 2020). Thus, the use of a single reference genome hampers our ability to study the full repertoire of genetic variation within a species (Golicz et al., 2016a; Zhao et al., 2018). Structural variants such copy-number variation can contain functional genomic elements that are usually under relaxed selective pressures and can serve as the basis of adaptation given specific environments and selective regimes (Lye and Purugganan, 2019). Coincidentally, copy-number variation and other structural variants play an important role in the emergence of domestication traits, as well as diversification traits in landrace varieties (Lye and Purugganan, 2019). Some studies estimate that at least one third of the known domestication loci are structural variants, and up to one in seven genes can be hemizygous (*i.e*., with one copy) in grapevine individuals (Zhou Y. et al., 2019). Despite its importance, structural variants cannot be properly analyzed using any of the aforementioned techniques. This led the research community to adopt the concept of the pan-genome, an idea that first appeared in microbiology (Tettelin et al., 2005), into the study of plant and animal genomes (Golicz et al., 2016a).

The concept of pan-genome rests on the idea that the genomes of individuals within a population or species share a core set of genes that unifies them (i.e., the core genome), but also contain a fraction of genes that are absent from one or more individuals (i.e., the accessory or dispensable genome), which altogether give rise to the pan-genome of such population or species (Tettelin et al., 2005).

There are three main methods to generate a pan-genome: the alignment and comparison of multiple *de novo* genome assemblies, the iterative assembly of several genomes from an initial reference or the use of *de Bruijn* graph assemblers to jointly assemble several genomes (Golicz et al., 2016a; see Table 1). Since domestication reduces the genetic diversity of a taxon, often eliminating portions of the dispensable genome that contain genes involved in local adaptation, the use of wild relatives is crucial to generate a representative pan-genome for a species (Khan et al., 2020). Once a pan-genome is generated, it can be used alongside whole-genome sequencing data to analyze the structural variants between and within populations, revealing novel loci involved in the development of domestication-related traits that would have stayed hidden when using a single reference genome (Li et al., 2014; Zhao et al., 2018). Besides, the use of a pan-genome alleviates the inherent reference biases of a single reference genome (Günther and Nettelblad, 2019).

**FIGURE 2:**
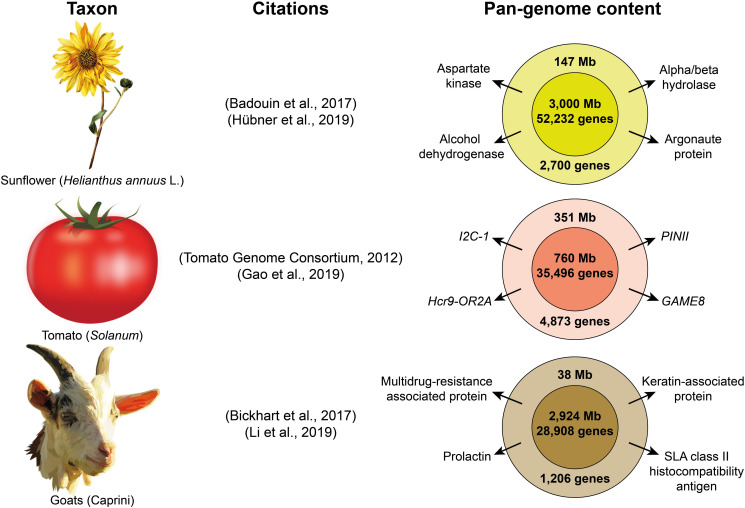
Examples of three pan-genomes of domesticated taxa. The citations included correspond to the publications of the original reference genomes and the subsequent pan-genome assemblies. The inner circles on the right represent the content of the reference genome, while the outer circles represent the additional nonreference content retrieved with the pan-genome. Some examples of important genes are show for the nonreference part of each pan-genome. (images obtained from Openclipart).

Pan-genome studies have revealed additional selective sweeps and structural variants associated to the domestication process, which were not identified using sequencing data with a single reference genome (Li et al., 2014; Zhao et al., 2018). Pan-genomes are already available for several species (Figure 2) such as maize (Brohammer et al., 2018), wheat (Montenegro et al., 2017), *Brassica oleracea* (Golicz et al., 2016b) or *Brassica napus* (Hurgobin et al., 2018); and pan-genome analyses to study domestication have already been performed in soybean (Li et al., 2014), rice (Zhao et al., 2018), sunflower (Hübner et al., 2019) and tomato (Gao et al., 2019). While current eukaryote pan-genome analyses are focused on plant species (Golicz et al., 2016a, see Table 1) and goats (Li et al., 2019), other livestock researchers may soon venture into this field. As sequencing technologies become cheaper, multiple pan-genomes from different species of the same genus should eventually be combined to create a super-pan-genome that represents the entire genetic content available in a genus with one or more domesticated taxa, as it would include the diversity of all their wild relatives (Khan et al., 2020).

## Population Genetics and Demographic Analyses of the Domestication Process

Demography and population size changes during the domestication process is tightly related to unraveling some of the most fundamental questions of the domestication process. These analyses can help answer questions such as possible centers of origin and diversification, patterns of migration and expansion throughout these centers, gene flow between domesticated and wild taxa, number of domestication events, the extent of genetic erosion in the domesticated taxon, levels of global genetic differentiation between wild and domesticated taxa, the patterns of adaptive and neutral introgression among them, and in some cases even the number of generations that have elapsed since domestication and other processes such as differentiation and local adaptation of domesticated taxa (Meyer and Purugganan, 2013; Guerra García and Piñero, 2017).

### Genetic Diversity in Populations

A first necessary step for the SNP data is to extract and compare the summary statistics of population genetics within and between populations (Andrews et al., 2016). This information describes the genetic diversity in populations, including the estimate of allele frequencies (usually denoted as *p* or the frequency of the most abundant allele), observed heterozygosity (*H*_O_), expected heterozygosity (*H*_E_), nucleotide diversity (*π*), number of segregating sites (*S*) and number of private alleles (*i.e*., alleles only found in one population). These summary statistics can reveal the level of genetic erosion in domesticated plants and animals when compared to the ancestral wild population, which is expected due to severe bottlenecks, selective sweeps and inbreeding (Groeneveld et al., 2010; Gepts, 2014). One should be aware that reference bias can influence the relative genetic variation observed between the wild and domesticated populations, which could be alleviated using more than one reference or using a pan-genome (Günther and Nettelblad, 2019).

### Population Structure

It is also important to describe the population structure (*i.e*., the genetic differentiation among populations) of domesticated taxa and of their wild relatives, as it can reveal the influence of historical events that shaped the genetic diversity of the organisms (Linck and Battey, 2019). The level of population structure between wild and domesticated taxa can be determined by several factors, such as the number of generations since domestication started, the intensity of the selective pressures imposed to the domesticated taxon, the intensity of the bottlenecks suffered though the domestication process, and the frequency of gene flow between the domesticated taxon and its wild relative (Meyer and Purugganan, 2013).

The *F*-statistics are classic estimates of population genetics that are based on the heterozygosity values within and among populations, which can reveal patterns of inbreeding, gene flow and differentiation between and within populations (Andrews et al., 2016). Of these, the *F*_ST_ statistic is of particular interest, since it can be used to detect population structure between wild and domesticated populations, or between different domesticated varieties (Andrews et al., 2016). These estimates are relatively simple to calculate, but they require *a priori* assignment of individuals to discrete populations, which may be wrongly assigned, may not reflect natural populations or may simply be unknown (Linck and Battey, 2019).

Methods based on population clustering have become popular for describing genetic structure, as they do not require *a priori* population assignment. These clustering methods can be classified into parametric and non-parametric methods (Linck and Battey, 2019). Parametric methods, also known as model-based methods, assign individuals into a predefined number of *K* populations based on their genotypes and the allele frequency of each locus (Pritchard et al., 2000). Several parametric methods have been described that successfully analyze genomic datasets to infer population structure (*e.g.*, Tang et al., 2006; Alexander et al., 2009; Raj et al., 2014), but one has to be careful when using them, as they assume linkage equilibrium and Hardy-Weinberg equilibrium in the dataset (Linck and Battey, 2019), so SNPs should be filtered accordingly before these methods can be confidently used (Wigginton et al., 2005; Mathew et al., 2018). Furthermore, parametric methods have been found to be susceptible to changes in the SFS generated by minor allele frequency thresholds that are commonly used to filter population genomics data because low-frequency polymorphisms are expected to contain information about recent events, which adds uncertainty to the assignation of individuals in populations that reflect ancient demographic events (Linck and Battey, 2019).

Non-parametric methods include principal component analyses, discriminant analyses of principal components and *K*-means clustering. These methods define populations and genetic structure by transforming the genetic data into uncorrelated variables – named eigenvectors or principal components – to identify groups within the dataset (Patterson et al., 2006; Jombart et al., 2010; Linck and Battey, 2019). Non-parametric methods were designed to work with large amounts of genomic data (Patterson et al., 2006; Jombart et al., 2010) and they are more robust to changes in the SFS than the parametric methods, so it is recommended to run both types of methods and compare their results before making further inferences (Linck and Battey, 2019).

### Inferences in Changes of Population Sizes Throughout Time

An important aspect of the demographic history of domesticated taxa is the analysis of the change in the effective population size (*N*_e_) in the populations throughout time (Chen J. et al., 2018). The concept of *N*_e_ reflects the estimated populations size in a Wright-Fisher model given an observed genetic variation, so these estimations hardly reflect the census population size of real populations (Charlesworth, 2009), and can also be affected by reference biases and allele dropouts. Changes in *N*_e_ can reveal or at least hint on the demographic history of taxa throughout the domestication process, such as expansions or bottlenecks. These changes can help to understand other evolutionary aspects of domestication concerning natural and artificial selection, such as the efficiency of selection and the accumulation of deleterious mutations in domesticated taxa (Chen J. et al., 2018; Allaby et al., 2019).

The domestication process is expected to include a bottleneck as a consequence of subsampling the genetic diversity in the wild ancestor, followed by a population expansion as domesticated taxa diversify (Meyer and Purugganan, 2013), although this idea has been recently challenged by paleogenomic studies (Allaby et al., 2019). Many methods exist to explore the changes in *N*_e_ throughout time, whose approach sometimes depends on the type of data available. It should be noted that all the methods to infer historical changes in *N*_e_ are susceptible to predicting false bottlenecks when populations are structured, so as indicated above, genetic structure should be evaluated and properly accounted for (Nielsen and Beaumont, 2009).

Studies with few individuals and high sequencing depth may use the Pairwise Sequentially Markovian Coalescent model (PSMC; Li and Durbin, 2011) or the Multiple Sequential Markovian Coalescent model (MSMC; Schiffels and Durbin, 2014) to analyze the demographic history of domesticated and wild taxa. The PSMC and MSMC models can infer changes in *N*_e_ throughout time (bottlenecks and expansions) by calculating the distribution of the time of coalescence between all the heterozygous loci in complete diploid genomes (Li and Durbin, 2011; Schiffels and Durbin, 2014). These models can also calculate the time of coalescence (*i.e.*, separation, and in some cases the domestication time) between two genomes given a specified mutation rate, recombination rate and generation time (Li and Durbin, 2011).

However, the genomes used in PSMC or MSMC must be of very good quality, having an average sequencing depth of the very least 18x, at least 10 reads per site, and less than 25% of missing data (Nadachowska-Brzyska et al., 2016). Besides, PSMC has several limitations compared to other estimators of *N*_e_ and is particularly susceptible to predicting false bottlenecks when populations are structured (Mazet et al., 2015). Nevertheless, this can be properly handled by comparing models of instantaneous *N*_e_ size change against models of classical symmetric islands using a maximum-likelihood approach (Mazet et al., 2015).

Multiple Sequential Markovian Coalescent can infer more recent changes in *N*_e_ compared to PSMC (Schiffels and Durbin, 2014), so it may be convenient to explore recent demographic expansions in diversified domesticated taxa (Allaby et al., 2019). For example, MSMC was used to infer population bottlenecks in East Asian and Western Eurasian dogs, as well as divergence times between wolves and dogs around 60,000–20,000 years ago (Frantz et al., 2016), while PSMC was used to determine a severe bottleneck in African rice around 15,000–13,000 years ago (Meyer et al., 2016).

Other methods rely on population data at a genomic scale from many (sometimes hundreds) individuals (as obtained from exome sequencing or RAD-seq), namely the extended Bayesian skyline plots (Heled and Drummond, 2008; Trucchi et al., 2014) and the stairway plots (Liu and Fu, 2015). Since *N*_e_ is a crucial concept in coalescent theory, extended Bayesian skyline plots and stairway plots rely on the SFS calculated from the population data to estimate *N*_e_ (Heled and Drummond, 2008; Liu and Fu, 2015). The inferences made from these two methods are comparable to those obtained from PSMC and MSMC, although they rely on different kinds of datasets (Liu and Fu, 2015). Furthermore, stairway plots are more efficient in inferring recent demographic history, whereas PSMC is more reliable for ancient demographic events (Liu and Fu, 2015).

### Estimating Gene Flow and Introgression Between Populations

Ancient gene flow and local ancestry (*i.e*., the genetic ancestry of an individual for an specific chromosomal position; Thornton and Bermejo, 2014) are also important aspects of plant and animal domestication that need to be addressed, since they can describe the genetic contribution of different ancestral populations in the genomic architecture of extant populations, such as wild and domesticated taxa (Price et al., 2009; Pickrell and Pritchard, 2012).

One approach to assess ancient gene flow are graph-based methods that incorporate the possibility of ancient gene flow between distantly related populations (Pickrell and Pritchard, 2012). This type of methods represents the relationships between populations as a bifurcating tree, where internal nodes can also be interconnected forming a graph that represents ancient gene flow that contributed to modern genetic variation (Pickrell and Pritchard, 2012). For example, graph-based analyses have revealed constant gene flow between sympatric populations of domesticated and wild pearl millet (Burgarella et al., 2018), constant gene flow between domesticated and wild pigs (Frantz et al., 2015) but lack of hybridization events between wild and domesticated populations of goats and sheep (Alberto et al., 2018).

Another popular test to infer ancient admixture is the ABBA-BABA test, also known as the *D*-statistic, which evaluates the allelic patterns of three taxa and compares them to an outgroup to identify genomic regions with an excess of shared derived variants that are not concordant to the species tree (i.e., ABBA-BABA patterns), which suggest introgression events (Durand et al., 2011). The f${}_{\text{d}}^{\wedge }$ test, which is derived from the *D*-statistic, can help discriminate between introgression events and nonrandom mating in ancestral structured populations (Martin et al., 2015). The *D*-statistic is sensitive to both introgression and incomplete lineage sorting, so both signals can be separated by testing deviations in the symmetry of branch lengths between the gene trees and the species tree (Edelman et al., 2019). By the same logic, the *D*_3_ test can also infer introgression events by analyzing the symmetry in branch lengths, without the need for an outgroup (Hahn and Hibbins, 2019). The *D*-statistic has been used to infer several introgression events between species of the *Bos* genus during domestication (Wu et al., 2018).

On the other hand, local ancestry methods can reveal which chromosomal segments in the genome were inherited from different ancestral source populations (Price et al., 2009). These methods use the data obtained from linkage disequilibrium between loci to assign ancestry in each portion of the genome in comparison to reference populations that depict ancestral source populations, requiring an *a priori* assignation of unadmixed reference populations in order to assign local ancestry to the populations of interest (Price et al., 2009). The analysis reveals chromosomic blocks that can be assigned to either a wild or a domesticated ancestry in hybrid populations, which may reveal historical processes of introgression and local adaptation in modern domesticated populations, as well as potential targets for selective breeding (Janzen et al., 2019).

Many methods exist that can infer local ancestry using genome-wide population data, and all of them require a high-quality reference genome (preferably assembled at a chromosome-level) in order to detect the ancestry of chromosomal segments (*e.g.*, Price et al., 2009; Baran et al., 2012; Maples et al., 2013; Dias-Alves et al., 2018). For example, a local ancestry analysis of East Asian domestic cattle revealed introgressed blocks inherited from ancient banteng and yak populations that contained genes enriched in sensory perception of smell, transmembrane transport and antigen processing (Chen N. et al., 2018).

### Using Demographic Simulations to Infer Domestication Scenarios

The previous descriptive tools can help us explore possible evolutionary and demographic scenarios in the absence of *a priori* hypotheses (Liu and Fu, 2015). However, for domesticated taxa we usually have additional classic botanical, zoological, morphological, paleoclimatic, archeological, ethnobiological and biogeographical data that may suggest some likely scenarios (Gerbault et al., 2014). Thus, demographic modeling can be used to test explicit demographic scenarios by comparing simulations of SFS in such scenarios to the observed data (Gerbault et al., 2014; Liu and Fu, 2015). There are many methods available for demographic modeling, which can be more suitable depending on the type of scenarios that need to be tested (Anderson et al., 2005; Gutenkunst et al., 2009; Excoffier and Foll, 2011; Cornuet et al., 2014). All these methods rely on some basic tenets of coalescent theory (Liu and Fu, 2015), so they are also susceptible to possible biases in the observed genetic variation in the populations.

For example, the approximate Bayesian computation (ABC) method compares the summary statistics of several simulated scenarios against the observed data to accept or reject certain demographic hypotheses (Cornuet et al., 2014; Gerbault et al., 2014). This method can help us determine certain parameters of our models and can be used with genome-wide datasets (Cornuet et al., 2014).

Other methods based on diffusion approximation can help us infer the demographic history of multiple populations and their interaction through migration and admixture using biallelic SNP data (Gutenkunst et al., 2009). Demographic modeling has helped test the number of domestication events as well as intercontinental migratory events in cattle (Pitt et al., 2019). Coalescent simulations have supported a common origin for all the domesticated varieties of pearl millet (Burgarella et al., 2018), while the ABC method has revealed that the most likely scenario in the domestication of the scarlet runner bean consists of a single domestication event around 21,000 years ago with a mild bottleneck effect (Guerra-García et al., 2017).

## Identifying Genes Under Selection During Domestication

Demographic processes are important to understand the general history that led to the domestication of plant and animal taxa, but many studies are specially interested in finding the selected genes that explain the phenotypic differences between domesticated taxa and their wild counterparts (Wang G.-D. et al., 2014; Kantar et al., 2017). Indeed, the detection of these genes under selection during domestication is critical to understand the genetic basis of domestication syndromes, especially for detecting genetic variation relevant for future improvement and selective breeding (Hufford et al., 2012).

When a genetic variant increases its frequency due to positive selection (*i.e*., selection favoring the fixation of a new allele), the adjacent alleles (*i.e*., physically connected in the same chromosomal region) also increase their frequency in a process known as hitchhiking (Smith and Haigh, 2007). Once the genetic variant under selection reaches a high frequency or fixation, the hitchhiking effect reduces or even eliminates the genetic variation around the selected locus, producing what is known as a selective sweep (Vitti et al., 2013; Pavlidis and Alachiotis, 2017). The size and intensity of a selective sweep depends on the rate of recombination in the genome, and on the intensity of the selective pressure (Smith and Haigh, 2007), which may be weaker in conscious selection compared to some cases of natural selection (Fugère and Hendry, 2018; Yang et al., 2019). Luckily, the signals of a selective sweep can be detected when the selection event occurred “recently” in an evolutionary timescale, as it is the case for domestication (Vitti et al., 2013).

Different bottom-up methods using population genomics data have been developed to detect the regions in the genome that were selected for during domestication, which we will refer to as candidate loci. We can mention methods for detecting regions with higher population differentiation compared to the rest of the genome, methods for detecting local changes in the SFS throughout the genome, and methods that detect extended regions with strong linkage disequilibrium compared to other haplotypes in the genome (see Supplementary Table S1 for a summary of methods to detect selective sweeps). Alternatively, a GWAS can be performed to detect the association of a genetic variant to a specific phenotype of interest (Wang G.-D. et al., 2014).

### *F*_ST_ Outlier Tests to Detect Candidate Genes

Besides the standard use of *F*_ST_ to detect global population structure, the *F*_ST_ statistic can also be used to detect signals of selective sweeps between populations, namely between wild and domesticated taxa (Gepts, 2014). While a global *F*_ST_ statistic (involving all the analyzed loci or SNPs) can reveal the overall genetic structure between populations, a local *F*_ST_ statistic calculated for each locus or SNP along the genome can evaluate whether particular regions of the genome are more differentiated from what is expected due to demographic processes, which can be interpreted as signals of a selective sweep (Nei and Maruyama, 1975). Many different methods exist that are based on the *F*_ST_ statistic, which are collectively known as *F*_ST_ outlier tests (Foll and Gaggiotti, 2008; Excoffier et al., 2009; Bonhomme et al., 2010; de Villemereuil and Gaggiotti, 2015; Lotterhos and Whitlock, 2015), that differ mainly on the underlying model used to calculate the null distribution of the *F*_ST_ values, and thus its ability to detect outliers (Supplementary Table S1).

*F*_ST_ outlier tests are able to detect selective pressures following a bottom-up approach, but their efficiency is determined by a multitude of factors that should be carefully accounted for before using them, such as the sampling scheme used to obtain the population data, the total size of the dataset (*i.e*., number of populations, of individual per population and of SNPs analyzed), the intensity of the selective pressure, the selfing or allogamous nature of its sexual reproduction, and the migration patterns and genetic structure among populations (De Mita et al., 2013; Lotterhos and Whitlock, 2014, 2015).

Some successful examples in the use of *F*_ST_ outlier tests include the detection of domestication candidate genes in apple involved in fruit development, size, acidity and sugar metabolism (Khan et al., 2014), the finding of candidate domestication genes involved in metabolism and oil biosynthesis in sunflower (Baute et al., 2015), the description of candidate diversification genes between pig breeds associated to the shape of the skull (Wilkinson et al., 2013), and the identification of candidate loci between wild and domesticated salmon strains involved in body weight, condition factor, male maturation and a brain related protein (Vasemägi et al., 2012).

### Site Frequency Spectrum Based Tests to Detect Selective Sweeps

Selective sweeps alter the SFS that would be expected under neutral evolution processes because of the reduction in the genetic diversity around the loci under selection (Vitti et al., 2013). The genomic region under selection skews the SFS into an excess of high frequency derived alleles when the selective sweep was recent, since the alleles that were linked to the favored selected locus also reach high frequencies (Fay and Wu, 2000). However, after all the high-frequency alleles reached fixation, the genomic region under the selective sweep will have little to no variation, while mutations will slowly generate new allelic variants, skewing the SFS into an excess of low frequency variants (Zeng et al., 2006). Several tests have been developed to detect skews in the SFS, each of them capable of detecting changes in different parts of the SFS (Supplementary Table S1), making them complementary to one another (Zeng et al., 2006; Vitti et al., 2013).

Even though SFS based tests are powerful tools to detect selection, it is important to remember that the SFS at the global genomic scale is also altered by demographic events such as bottlenecks that produces an excess of low frequency variants, and expansions that generates an excess of intermediate frequency variants (Vitti et al., 2013). Thus, it is mandatory to have a previous prediction of the demographic history of the populations in order to properly adjust the null hypothesis in each test (Ross-Ibarra et al., 2007).

The well-known summary statistic called Tajima’s *D* is sensitive to changes in low-frequency variants, making it particularly useful to detect selective sweeps before and after the selected locus reaches fixation, although low-frequency variants can also be observed in loci under purifying selection (Tajima, 1989; Zeng et al., 2006). Tajima’s *D* is also sensitive to intermediate-frequency alleles, making it useful to detect balancing selection (Tajima, 1989) or even some forms of soft selective sweeps generated by standing genetic variation (Prezeworski et al., 2005).

Conversely, Fay and Wu’s *H* is sensitive to changes in high-frequency variants, which are only altered by positive selection, making it very useful when used alongside Tajima’s *D* (Fay and Wu, 2000). Unlike Tajima’s *D*, Fay and Wu’s *H* needs an outgroup species in order to differentiate ancestral alleles from derived alleles and thereby to know whether the derived alleles are at high or low frequencies (Fay and Wu, 2000).

Zeng et al. (2006)’s *E* is sensitive to both low and high frequency variants, making it particularly powerful to detect selective sweeps before or after the selected locus reached fixation, also needing an outgroup in order to differentiate derived alleles from ancestral alleles).

There are some tools available to implement SFS based tests using genome-wide data, that can perform all the above tests (*i.e.*, Korneliussen et al., 2013, 2014; Rozas et al., 2017). For example, Tajima’s *D* test was used alongside other methods to detect selective sweeps associated to the domestication of yaks (Qiu et al., 2015), Zeng’s *E* test helped discover 125 selective sweeps associated to the domestication of horses (Librado et al., 2016), and the complementary implementation of Tajima’s *D*, Fay and Wu’s *H* and Zeng’s *E* revealed several candidate genes that share similar functions between peach and almond (Velasco et al., 2016).

The reduction of diversity (ROD) test is another popular SFS-based method to detect selective sweeps that has been particularly useful for the study of domestication (Supplementary Table S1). ROD compares local *π* values of domesticated taxa against the local *π* values of its wild relatives, using sliding windows alongside the genome (Guo et al., 2012; Huang et al., 2012; Qi et al., 2013; Schmutz et al., 2014). The ROD method has been used to successfully detect candidate domestication genes in rice (Huang et al., 2012), watermelon (Guo et al., 2012), cucumber (Qi et al., 2013), common bean (Schmutz et al., 2014), and chickpea (Varshney et al., 2019), to name a few.

### Linkage Disequilibrium (LD) Based Methods to Detect Selection

Given that selective sweeps remove the variation in regions adjacent to the locus under selection, they can form haplotype blocks that extend in strong LD compared to other haplotypes in the same locus because they reached a medium-to-high frequency in the population swift enough so they are not yet disrupted by recombination (Sabeti et al., 2002; Vitti et al., 2013). This pattern has been exploited to develop several methods based on LD to detect selective sweeps of recent origin (Vitti et al., 2013). Interestingly, LD-based methods are sensitive enough to detect both strong and soft selective sweeps (Garud et al., 2015), as well as partial or incomplete selective sweeps (Vitti et al., 2013), making them excellent tools to study recent and ongoing selection events, such as those occurring during domestication and the subsequent diversification of landraces (Supplementary Table S1).

Since the above rationale relies on LD decay due to recombination, any method based on LD requires to control for local variation in recombination rates in order to reduce false positives (Sabeti et al., 2002). The extended haplotype homozygosity (EHH) is a widely used statistic in LD-based methods that is defined as the probability that two orthologous genomic regions carrying a “core” haplotype of interest (*i.e*., the part of the haplotype that is shared by all the individuals carrying it, such as the allele under positive selection) in the population are identical by descent (*i.e.*, they were inherited by the same ancestor), as one looks to a specified distance farther away from the core region (Sabeti et al., 2002).

Among the LD based methods that uses the EHH, we can mention the long-range haplotype (LRH) test, sometimes named the relative EHH (rEHH) test, which controls for local recombination rates by comparing the EHH of several haplotypes localized within the same locus (Sabeti et al., 2002). Other EHH based methods include the whole-genome long-range haplotype (WGLRH) test that uses sliding windows to perform the LRH test (Zhang et al., 2006), the long-range haplotype similarity (LRHs) test (Hanchard et al., 2006), the integrated haplotype score (iHS) which is particularly sensitive to incomplete selective sweeps and soft sweeps (Voight et al., 2006) and the cross-population extended haplotype homozygosity (XP-EHH) statistic that is able to detect selective sweeps after the selected allele reached fixation (Sabeti et al., 2007). The iHS and the XP-EHH statistics can be regarded as complementary to each other, enabling the detection of incomplete and complete selective sweeps in the target population (Vitti et al., 2013).

All the LD-based tests that make use of the EHH statistic require the previous phasing of the chromosomes in order to work (*i.e*., assignation of alleles in an individual to their corresponding maternal and paternal haplotypes), which may or may not be possible depending on the sequencing depth and type of data available for the analysis (Delaneau et al., 2013). For instance, a reference genome is usually needed in order to phase genotypes, since most methods rely on the information of proximity between alleles and their distribution within individuals in a population to assign haplotypes (Delaneau et al., 2013) although new methods are emerging that can phase genotypes without a reference genome (Money et al., 2017).

There are other LD-based methods that do not make use of the EHH statistic, such as the LD decay (LDD) test, which rely on individuals that are homozygous for any given SNPs to look for LD differences between alleles in a population (Wang et al., 2006) or the ω statistic that scans for high SNP correlation coefficients around a site under selection (Kim and Nielsen, 2004; Alachiotis et al., 2012). Another method that do not require chromosome phasing is the regression-based test, which relies on the reduction of heterozygosity as one approaches the locus under selection in a genome to infer selective sweeps (Wiener and Pong-Wong, 2011). Other LD-based methods exploit the estimation of identity-by-descent using genome-wide data to detect haplotypes that are shared between several unrelated individual (> 10 generations) to infer selective sweeps without previous knowledge of the pedigree of individual (Han and Abney, 2013), so they might prove useful to study recent domestication processes.

Some examples of LD-based methods used to explore the domestication process includes an analysis using LRH to detect signatures of selection associated to dairy and beef cattle breeds (Bomba et al., 2015), a study using the XP-EHH statistic to find signals of selective sweeps in Jinhua pigs (Li et al., 2016), and a paper focused on the diversification of goat landraces that calculated the iHS and the XP-EHH statistics alongside other tests to detect selective sweeps between goat breeds (Bertolini et al., 2018).

Other important tests include the XP-CLR test (Chen et al., 2010) and the μ statistic (Alachiotis and Pavlidis, 2018) which implement multiple signatures to detect selective sweeps (Supplementary Table S1) and have been used to detect candidate loci in maize and African rice, respectively (Hufford et al., 2012; Ndjiondjop et al., 2019).

### Using GWAS to Detect Domestication-Associated Loci

Genome-wide association studies have been used extensively to uncover the genetic variants that underlie domestication traits (Shi and Lai, 2015). The domestication traits that can be analyzed through a GWAS can encompass any biological characteristic from simple morphological traits (Jiao et al., 2012) to the production of certain metabolites (Shang et al., 2014), tame behavior in animals (Ilska et al., 2017), resistance or susceptibility to certain diseases (Wang et al., 2012), or adaptation to certain environmental conditions (Song et al., 2018).

An important advantage of the GWAS over the bottom-up approaches is its ability to detect polygenic effects on single traits of interest, which is commonplace considering that genes interact between them and the environment to generate phenotypes (Gibson, 2018).

A prerequisite before preforming a GWAS is to have large sample sizes in both the number of sequenced genetic variants and the number of individuals included in the study, as they are necessary to obtain the statistical power to detect variants with small effects and to reduce the risk of false positives (Wang G.-D. et al., 2014).

Some recent examples include the use of a GWAS to identify candidate genes with unknown functions involved in several agronomic traits, including drought and heat tolerance in chickpea (Varshney et al., 2019); a GWAS that revealed loci associated to fruit size and quality in peach (Cao et al., 2019); and a GWAS that uncovered the genetic variants involved in the absence of anthocyanin in domesticated rice compared to its wild relative (Zheng et al., 2019).

## Ancient DNA and Paleogenomics of Domesticated Taxa

Extant domesticated taxa lack the information of ancient genetic diversity that was lost through bottlenecks, selection and genetic drift (Ramos-Madrigal et al., 2016). However, the analysis of ancient DNA can allow the research community to overcome some of these limitations (Irving-Pease et al., 2019). Ancient DNA retrieved from archeological sites allows the study of the rate at which domestication happened, as well as revealing which genes were important at the beginning of this process (Vallebueno-Estrada et al., 2016; Irving-Pease et al., 2019). Thus, paleogenomics is becoming a novel research area for understanding the process of plant and animal domestication (Irving-Pease et al., 2019).

### Extraction and Sequencing of Ancient DNA

An important limitation of paleogenomic analyses is the level of preservation of the ancient DNA itself, as well as the total yield of extracted DNA (Sawyer et al., 2012). The DNA molecules that are extracted from tissues that are not conserved on permafrost and are older than 100 years are usually shorter than 100 bp (Sawyer et al., 2012). The strand breaks of such fragments are also non-random, as purines are enriched before the strand breaks (Sawyer et al., 2012). Additionally, these fragments incorporate cytosine-to-uracil mutations on their ends, further hindering the analysis of the sequenced fragments (Sawyer et al., 2012). Even though these characteristics hamper the sequencing and analysis of ancient DNA, they are also useful to differentiate between real ancient DNA and extant DNA contamination (Sawyer et al., 2012). Furthermore, due to the scarce ancient material located throughout few archeological sites worldwide, sample sizes in paleogenomic studies are very small, usually one or few individuals per location and sometimes only one locality (*e.g.*, Wales et al., 2016; Ramos-Madrigal et al., 2016).

Given the above difficulties and the uniqueness of the biological material retrieved from archeological sites, it is crucial to extract and sequence as much ancient DNA as possible while avoiding DNA contamination (Gamba et al., 2016). Major efforts have been made to develop efficient protocols for ancient DNA extraction (Gamba et al., 2016) and single-strand library preparation for high-throughput sequencing (*e.g.*, Gansauge et al., 2017). Organelle genomes were usually the target for ancient DNA sequencing because multiple copies of these can be found within each plant and animal cell and can reveal several demographic processes (Wales et al., 2016; Irving-Pease et al., 2019). Nonetheless, more evolutionary information can be retrieved from nuclear DNA, which is the main target for modern paleogenomic studies (Wales et al., 2016; Irving-Pease et al., 2019).

### Insights of Paleogenomic Data in Domestication

Paleogenomic studies are challenging some of our previous ideas of the domestication process, such as the occurrence of ancient domestication bottlenecks, which appear to be absent in several archeological plant genomes, suggesting that the reduced diversity in domesticated taxa may be a more gradual process from what was expected using DNA of extant populations (Allaby et al., 2019). For example, several archeological samples of *Sorghum bicolor* from different time periods (ranging from 1800 to 100 years ago) were compared to extant individuals of the species, revealing that this crop did not suffered an initial domestication bottleneck, but rather that the reduction in genetic diversity, and its associated mutational load, occurred gradually throughout time (Smith et al., 2019).

Paleogenomics is also revealing important aspects of plant and animal domestication, such as the first genetic steps towards domestication syndromes as well as the overall graduality of the process (Ramos-Madrigal et al., 2016; Vallebueno-Estrada et al., 2016; Daly et al., 2018). For example, archeological remains of goat populations have revealed multiple domestication processes in ancient wild goats, possible dispersal routes of ancient goat populations and signs of early selective pressures towards candidate genes involved in pigmentation, milk production, size, reproduction and changes in diet (Daly et al., 2018). Likewise, several archeological maize samples retrieved from the Tehuacán Valley in Mexico have revealed that early domesticates already presented signals of selective sweeps on important candidate genes, such as *teosinte branched1* and *brittle endosperm2*, but lacked selective sweep signals in other important candidate genes present on modern maize populations, even though these ancient maize populations were already endogamous and more closely related to modern maize than to wild teosinte, revealing that maize domestication was a gradual process ranging thousands of years (Ramos-Madrigal et al., 2016; Vallebueno-Estrada et al., 2016).

Other examples demonstrate the importance of paleogenomic studies in domesticated taxa, including grapevine (Wales et al., 2016), barley (Mascher et al., 2016), sunflower (Wales et al., 2019), horses (Schubert et al., 2014), dogs (Frantz et al., 2016) and cats (Ottoni et al., 2017).

## RNA Sequencing to Detect Differentially Expressed Genes Associated to Domestication

Besides the use of RNA-seq to obtain population-level data, comparative transcriptomics is a good way to find or support the validity of candidate genes (Hekman et al., 2015). Transcriptomic analyses between domesticated and wild taxa can reveal important changes in gene expression associated to domestication (Koenig et al., 2013; Hekman et al., 2015; Hradilová et al., 2017). Likewise, the analysis of hybrids between domesticated and wild individuals can reveal important patterns of allele-specific regulation and the role of *cis*/*trans* regulatory elements in the emergence of domestication traits (Bell et al., 2013; Lemmon et al., 2014).

### The Experimental Design of Differential Expression Analyses

Transcriptomic profiles are tissue-specific and time-dependent (Hekman et al., 2015). Thus, a good experimental design can reveal important loci involved in the phenotypic differences associated to domestication syndromes, such as suppression of secondary metabolites, changes in form, size, taste, absence of defense mechanisms, seed dormancy, docile behavior, among other traits (Hekman et al., 2015). This can be done by comparing the total RNA expression of the tissue or organ of interest (Koenig et al., 2013), as well as comparing RNA expression throughout the developmental stages of such tissue or organ (Hradilová et al., 2017).

Since transcriptomic analyses are experimental by nature, experimental designs require biological replicates for each treatment, condition or organ to assess the variability in the data; as well as controlled environmental conditions to reduce possible biases and sources of error (Fang and Cui, 2011; Schurch et al., 2016). Empirical studies recommend using at least six biological replicates for each condition in the experiment, even though the use of three replicates is common, but discouraged (Burden et al., 2014; Schurch et al., 2016). Additionally, it is important to avoid committing errors in the experimental design that can bias the results of the RNA-seq experiment, such as using different sequencing technologies for each sample, using different methods for library preparations throughout the samples, sequencing each treatment in a different sequencing flowcell or different lanes within a flowcell (Fang and Cui, 2011). Other technical biases associated to adapter ligation and within-lane variation can be properly assessed when using biological replicates (Fang and Cui, 2011; see Table 1).

RNA-seq data can also be complemented with metabolomic data to infer the association between the differential expression of genes and the presence/absence of metabolites between wild and domesticated taxa (Hradilová et al., 2017).

After obtaining high-quality data with an appropriate experimental design, RNA-seq analyses usually follow a similar workflow, which should culminate in the detection of differentially expressed genes between a wild plant and its domesticated counterpart (Yang and Kim, 2015; see Table 1). These differentially expressed genes are most likely candidates that may explain to some degree the changes associated to domestication (Koenig et al., 2013; Hradilová et al., 2017). Nonetheless, one must be careful while interpreting the results of these studies, as some differentially expressed genes between wild and domesticated taxa may be a consequence, rather than a cause, of the domestication traits under study (Albert et al., 2012).

### Successful Examples of RNA-seq Experiments to Understand Domestication

RNA-seq analysis has been successfully employed to discover differentially expressed genes involved in the domestication of several plant species. For example, RNA-seq analyses between maize and teosinte found 600 differentially expressed genes and 1,100 genes with altered patterns of co-expression, mainly involved in biotic stress responses, and many of which were previously found as candidate genes using selective scans (Myers et al., 2012). Similar results have been found in tomato (Koenig et al., 2013), pea (Hradilová et al., 2017), common bean (Singh et al., 2018), and carrot (Machaj et al., 2018). This approach has also led to the discovery of differentially expressed genes between dogs and wolves associated to tameness (Li et al., 2013), as well as changes related to the immune system and aerobic capacity (Yang et al., 2018). Another study found differential isoform expression between wild and domesticated sorghum accessions, revealing that domestication can alter the patterns of alternative spicing (Ranwez et al., 2017). Hybrid studies have been performed between maize and teosinte, suggesting potential selection on *cis* regulatory elements associated with changes in ear tissue and previously reported candidate genes (Lemmon et al., 2014). Another hybrid study in *Capsicum annuum* using network analyses revealed that loss of function in *cis* regulatory sequences lead to transcriptional changes in *trans* elements that are associated with fruit morphology (Díaz-Valenzuela et al., 2020).

## Modern Epigenomics and Methodological Strategies to Explore Domestication

Epigenetics is classically defined as the heritable mechanisms that regulate gene expression without direct modifications to the DNA sequence, namely DNA methylation, RNA methylation, covalent histone modifications and chromatin assembly states (Sakurada, 2010; Zhao et al., 2017). Epigenetic variants, sometimes called epialleles, are local differences in these epigenetic marks between individuals in a population, which can have similar dynamics to genetic variants (Weigel and Colot, 2012; Guo et al., 2015). Since epigenetic mechanisms underly the ability of organisms to respond to changing environmental conditions, some epigenetic marks associated to these responses are more susceptible to change due to environmental input, while other marks involved in cell differentiation, embryonic development and core cellular functions might be more stable (Turner, 2009).

Most of the domestication studies that explain phenotypic differences between wild and domesticated taxa focus on genetic variation. However, the study of epigenomics may explain some of the missing heritability in domestication traits (i.e., the gap between the heritability of a trait estimated by classic genetics and GWAS), the patterns of differentially expressed genes that do not have clear signs of selective sweeps, or even connect the causality between the genetic variation that was selected for during domestication and the resulting phenotypes (Schmitz et al., 2013; Trerotola et al., 2015; Janowitz Koch et al., 2016; Bélteky et al., 2018).

Epigenetic variation can be inherited from one generation to the next in a process known as trans-generational epigenetic inheritance, which has been documented in plants and animals (Heard and Martienssen, 2014), even though the overall importance of this trans-generational epigenetic inheritance in plant and animal evolution is still debated (see Table 1). Nevertheless, we consider that studying epigenetic patterns associated to transcriptional activity and phenotypic traits should help understand the emergence of domestication phenotypes (Bélteky et al., 2018). If epigenetic variants such as single methylation polymorphisms (SMPs) show complete transgenerational inheritance, they can even be analyzed using the theoretical tools of population genetics to detect selective sweeps (Schmitz et al., 2013; Janowitz Koch et al., 2016).

In a similar fashion to GWAS, the use of epigenome-wide association studies (EWAS) can also reveal the association of an epigenetic variant to a trait of interest in domesticated taxa (Feeney et al., 2014). The same precautions taken in transcriptomic data should also be taken for epigenomic data, since the patterns of epigenetic marks in organisms are tissue-specific, time-dependent and sensitive to environmental input, meaning that epigenomic data should be analyzed for specific organs or tissues of interest in a controlled environment (Jensen, 2015). This is particularly important for the epigenetic marks that respond to environmental input, since domesticated taxa and their wild relatives live under different environmental conditions. Growing both taxa under controlled conditions will alter the natural state of these marks, but will also help differentiate the heritable epialleles associated to domestication traits (Turner, 2009).

### Obtaining Population Data From Epigenetic Marks

The most studied epigenetic mark is DNA 5-methylcytosine, which refers to the DNA methylation in cytosines which are usually associated to transcriptional gene silencing (He et al., 2011). Cytosine methylome data can be obtained using high-throughput sequencing technologies alongside bisulfite sequencing (Meissner, 2005). Bisulfite sequencing consists in the deamination of unmethylated cytosines through a bisulfite reaction, converting them into uracil, which are encoded as thymine by sequencing technologies (Frommer et al., 1992). The comparison of sequenced DNA that was treated with bisulfite alongside sequenced DNA without treatment can discriminate between methylated and unmethylated cytosines in an organ, tissue or cell-type of interest (Frommer et al., 1992).

Reduced representation bisulfite sequencing (RRBS) is a high-throughput technique with a similar rationale to RAD-seq that enriches the sequencing of CG rich regions of the genome after the digestion of restriction enzymes (Meissner, 2005). This makes the RRBS technique a cost-effective option to analyze cytosine methylation patterns in mammals, since its cytosine DNA methylation happens at CG sites (Meissner, 2005; He et al., 2011). Plant cytosine methylomes should instead be analyzed through MethylC-seq, which consists of whole-genome sequencing and bisulfite treatment (Urich et al., 2015), as cytosine methylation can also happen in CHG and CHH sites in plant genomes (He et al., 2011). Cytosine methylation can also be detected using methylated DNA immunoprecipitation sequencing (MeDIP-seq), which consists in shearing the genomic DNA into small pieces followed by the immunoprecipitation of the methylated cytosines using antibodies that recognizes 5-methylcytosine and finally sequencing the DNA sequences with the methylated sites using standard high-throughput sequencing technologies (Weber et al., 2005).

Besides cytosine methylation, adenine has also been shown to be methylated in both plants and animals (N6-methyldeoxyadenosine), which cannot be detected using bisulfite sequencing (Luo et al., 2015). However, genomic regions with methylated adenines can be detected using N6-methyldeoxyadenosine immunoprecipitation sequencing (6mA-IP-seq), which uses the same rationale as MeDIP-seq but requires antibodies that specifically targets N6-methyldeoxyadenosine (Fu et al., 2015). PacBio and Nanopore sequencing technologies are known to be sensitive to DNA methylation, regardless of it being on a cytosine or adenine, so they are currently being used as powerful, albeit expensive tools to evaluate DNA methylation patterns in genomes (Gouil and Keniry, 2019).

Histone modifications refers to either posttranslational covalent modifications in histones (methylations, acetylations, phosphorylations, ubiquitylations, ADP-ribosylations, sumoylations, crotonylations, malonylations, succinylations) or the substitution of canonical histones by histone variants with different amino acid composition (Bowman and Poirier, 2015). These histone modifications determine the functionality of local genomic regions by changing the state of the chromatin either through its direct effects on the chemical interactions between DNA and histones or through the recruitment of chromatin remodeling complexes (Bowman and Poirier, 2015).

Chromatin immunoprecipitation sequencing (ChIP-seq) can be used to assess the genome-wide association between DNA regions and specific histone modifications (Schmidt et al., 2009). ChIP-seq consists in the initial fixation of DNA-protein interactions using formaldehyde followed by DNA fragmentation and subsequent enrichment of the target histone modification using magnetic beads coupled to antibodies in order to sequence the genomic regions where the histone modification is present (Schmidt et al., 2009). ChIP-seq can also be used to assess the interaction between any DNA-binding protein such as transcriptional factors and specific genomic regions (Schmidt et al., 2009).

### Epigenomic Studies Applied to Understand Domestication

The current epigenomic analyses regarding domestication have focused on DNA methylation patterns (Jensen, 2015; Ding and Chen, 2018), but some studies have also ventured into histone modification patterns (He et al., 2014). Recent efforts are trying to connect the discoveries of genomics and epigenetics to understand the evolution of tameness in domesticated animals (Jensen, 2015). A study using RRBS that compared the DNA methylation patterns between wolves and dogs revealed signals of natural selection acting on SMPs which are enriched in transposons and genes involved in the regulation of neurotransmitters, suggesting a dog-specific silencing of genes involved in behavior (Janowitz Koch et al., 2016). Similarly, a recent study using MeDIP-seq in red junglefowl populations that were bred to have either high or low fear to humans discovered genomic region that were differentially methylated in genes that were previously related to tameness (Bélteky et al., 2018).

Other studies focused on plant domestication have found differentially methylated sites associated to domestication syndromes (Song et al., 2017; Shen et al., 2018). A study using MethylC-seq found 519 differentially methylated genes between domesticated and wild cotton from which some of them are associated with the observed differences in flowering time and seed dormancy between the wild and domesticated taxa (Song et al., 2017). Another study using MethylC-seq found 4,248 differentially methylated regions between wild and domesticated soybean and 1,164 differentially methylated regions between domesticated and improved soybean (Shen et al., 2018). As expected, the differentially methylated regions in soybean had higher genetic diversity compared to the regions with evidence of selective sweeps that were previously found, and interestingly, 22.5% of the differentially methylated sites could be associated to a causal genetic variant (suggesting that these genetic variants were responsible for the observed epigenetic patterns), whereas the rest of the differentially methylated regions could be interpreted as genuine epialleles located within genes involved in carbohydrate metabolism (Shen et al., 2018).

## Experimental Validation of Candidate Genes

Once we have evidence of candidate genes involved in the domestication syndrome, the necessary next step to understand the genetic basis of domestication is to design *in vitro* systems, knock-out, knock-down or knock-in experiments that validate the involvement of such genes in the observed phenotypes (Zhang et al., 2017). This can be performed either by direct alteration of the genome in the organism of interest, by using RNA interference or by designing heterologous systems in a model organism (Boettcher and McManus, 2015). As an example, a knock-out experiment with backcrosses between domesticated and wild mice elucidated the role of some genes involved in behavioral changes associated to mouse domestication (Chalfin et al., 2014).

Previous knock-out and knock-in experiments were restricted to model organisms, but nowadays experimental validation of candidate genes can be supported via knock-out and knock-in experiments, using novel genome editing tools (*e.g*., Shalem et al., 2014; Hahn et al., 2017; Ueta et al., 2017). Genome-editing tools are already available for a broad range of taxa, including dozens of crop species, but developing a working system in non-model organisms can still be a difficult task that can take several months or even years to accomplish (Shan et al., 2020), so doing collaborative studies alongside experimental researchers is recommended. In this moment, the leading toolset to perform genome editing is the Clustered Regulatory Interspaced Short Palindromic Repeats (CRISPR) system alongside the CRISPR associated protein 9 (Cas9), commonly known as CRISPR/Cas9, which can be used to eliminate, introduce or replace specific segments of DNA within a targeted site in a genome (Cong et al., 2013). Another useful tool for genome editing is the Transcription Activator-Like Effector Nuclease (TALEN) technology, which has its own advantages in comparison to CRISPR/CAS9 (Zhang et al., 2017). RNA interference can also help in validating the function of candidate genes, although it is limited to knock-down experiments (Boettcher and McManus, 2015). Heterologous expression in model organisms is a cost-effective alternative to validate candidate genes (e.g., Schweiger et al., 2010), although this method overlooks the interaction networks that exist *in vivo* which are accountable for the emergence of phenotypes (Rodríguez-Mega et al., 2015).

Regardless the genome-editing tool of choice (Boettcher and McManus, 2015; Zhang et al., 2017), genome edition is proving its usefulness to validate the effect of candidate genes involved in domestication through the introduction of domesticated alleles on wild relatives and vice-versa (Zhou J. et al., 2019), which can prove that the gene is indeed involved in the appearance of the domesticated phenotype (Zhou J. et al., 2019). This can be performed in the same way as a usual knock-out or knock-in experiment, where the edited locus must be validated through PCR and Sanger sequencing, a PCR-RFLP analysis or using Western-blot in case of a protein knock-out (*e.g.*, Ueta et al., 2017). The expected result of these type of studies is to find a modified phenotype after editing a candidate locus, either a wild individual with a domesticated-like phenotypic trait or a domesticated individual with a wild-like phenotypic trait (Zhou J. et al., 2019).

Of course, the above studies will hardly reproduce a complete domesticated or wild phenotype, since genetic elements interact in complex regulatory networks, including other elements within the genome as well as epigenetic and environmental components (Rodríguez-Mega et al., 2015), but nonetheless will be useful to understand the role of those genes in the emergence of domesticated phenotypes.

Once the candidate genes are validated, genome-editing tools can also become useful to introduce desirable traits from wild relatives to its domesticated counterparts, a goal of great interest for crop improvement (Zhou J. et al., 2019) and currently used to accelerate plant breeding and to fine-tune desirable traits (Wolter et al., 2019). Furthermore, recent efforts are trying to domesticate plant crops *de novo* by inserting the desired domestication alleles into their wild relatives, generating crops with the desired domestication phenotypes but without the problems of low genetic variation and accumulation of deleterious mutations that are an inevitable consequence of regular domestication processes (Fernie and Yan, 2019).

## Conclusion and Perspectives

Plant and animal domestication can be studied using genomic, transcriptomic and epigenomic strategies, revealing the action of evolutionary, ecological and anthropogenic processes (Kantar et al., 2017). These tools can lead us beyond the description of the possible historical scenarios that shaped the domesticated species, since we can explore the effects of domestication on the transcriptomic activity of a species (Hekman et al., 2015), test the validity of candidate genes associated to domestication phenotypes (Zhou J. et al., 2019) and analyze epigenetic patterns associated to domestication traits (Jensen, 2015). Many domesticated taxa remain genetically unexplored, and as sequencing technologies become cheaper and more efficient, domestication genomics will soon be available for polyploids and species with huge genomes (*e.g.*, Edger et al., 2019).

Nonetheless, the modern study of domestication of plants and animals should still be multidisciplinary, since genetics only tells us part of the story (Larson et al., 2014). An extended synthesis framework should also be considered to understand domestication, as these new studies are helping us understand niche construction and the emergence of domesticated phenotypes (Piperno, 2017). Other potential lines of work remain to be addressed in domestication studies, such as the changes in the chromatin architecture (e.g., Concia et al., 2020), the use of comparative proteomic atlases (e.g., Jiang Y. et al., 2019) and the analysis of cell-type divergences during development using single-cell RNA-seq data (Arendt et al., 2016). The use of this multi-omic approaches will help us create and compare developmental atlases (e.g., Walley et al., 2016) between wild and domesticated taxa to understand how morphology diverged during domestication.

## Author Contributions

JB-R, DP, and LE wrote the manuscript. All authors contributed to the article and approved the submitted version.

## Conflict of Interest

The authors declare that the research was conducted in the absence of any commercial or financial relationships that could be construed as a potential conflict of interest.
