# Supplementary material for: Genomic, Transcriptomic and Epigenomic Tools to Study the Domestication of Plants and Animals: A Field Guide for Beginners
Source: Front Genet. 2020 Jul 15;11:742. doi: 10.3389/fgene.2020.00742 (PMC7373799; doi:10.3389/fgene.2020.00742)
Supplement: Supplementary file 1 [file Table_1.docx]

Supplementary Material

# Supplementary Figures and Tables

**TABLE S1 |** Some examples of bottom-up tests designed to detect selective sweeps in populations using genomic data and their underlying models (modified from Vitti et al., 2013). See the main text to find the references cited in this table.

| **Underlying model** | **Test name** | **Rationale** |
| --- | --- | --- |
| *F_ST_* outlier tests | BayeScan | This method estimates the correlation between allele frequencies (*i.e.*, the genetic structure caused by demographic events) by calculating a multinomial-Dirichlet distribution using a Markov chain Monte Carlo Bayesian approach to subsequently detect outlier loci. This method assumes an island model (*i.e.*, gene flow is equally frequent between each pair of populations) which can lead to false positives when used in populations with limited gene flow and strong population bottlenecks, as expected for many domesticated taxa (Foll and Gaggiotti, 2008). |
|  | Excoffier, Hofer and Foll (EHF) test | Uses coalescent simulations to calculate the distribution of genetic diversity within and between populations to obtain a null distribution of FST values and detect outliers. This method is optimized for hierarchically structured populations (*i.e.*, genetic flow is more frequent within populations and between populations belonging to the same group such as domesticated or wild taxa) by using a hierarchical island model in which populations are assigned to groups *a priori* (Excoffier et al., 2009). |
|  | T_F-LK_ statistic | This method calculates a kinship matrix between populations by generating a neighbor-joining population tree in order to obtain a null distribution to detect *F_ST_* outliers. The statistic seems to be robust to complex demographic scenarios, reducing the emergence of false candidate loci (Bonhomme et al., 2010). |
|  | BayeScEnv | Uses the same approximation as BayeScan to detect *F_ST_* outliers, but it also incorporates environmental cues to detect adaptation to local environment (de Villemereuil and Gaggiotti, 2015). |
|  | PCAdapt | This method calculates the underlying population structure using a principal component analysis and then detects candidate loci under selection using Mahalanobis distance. This method is particularly powerful when handling admixed individuals and hierarchical structure in the studied populations (Luu et al., 2017). |
| SFS based methods | Tajima's *D* statistic | Calculated from the comparison of the nucleotide diversity (π) against Watterson's Theta (θ_W_). When π < θ_W_, the region has an excess of low-frequency variants, suggesting purifying or positive selection, whereas when π > θ_W_, the region has an excess of middle-frequency variants, suggesting balancing selection or a soft selective sweep (Tajima, 1989). |
|  | Fai and Wu's *H* statistic | Calculated from the comparison of π against θ_H_ or θ_L_, which are estimators of theta weighted by the homozygosity of derived variants. When π < θ_H_, the region has an excess of high-frequency variants, a characteristic signature of selective sweeps (Fay and Wu, 2000). |
|  | Zeng *et al*.'s *E* statistic | Calculated from the comparison of θ_L_ against θ_W_, rendering it sensible to changes in high and low-frequency variants, which are signals of selective sweeps before and after the fixation of the locus under selection (Zeng et al., 2006). |
|  | Reduction of diversity (ROD) test | The selective sweeps associated to domestication will form a pattern where the domesticated taxon will have a significantly lower diversity in that region compared to the overall diversity in its genome, while the wild taxon will not show reduced genetic diversity in that locus (π_wild_ > π_domesticate_) (Guo et al., 2012). |
| LD based methods | Long-range haplotype (LRH) test | This test uses the EHH statistic to detect whether an haplotype is inherited throughout the population without its disruption by recombination, suggesting that such haplotype is under positive selection (Sabeti et al., 2002). |
|  | Whole-genome long-range haplotype (WGLRH) test | The WGLRH test performs the LRH test throughout the entire genome using sliding windows to detect EHH outliers (Zhang et al., 2006). |
|  | Long-range haplotype similarity (LRHs) test | Calculates the similarity between homologous haplotypes by calculating an haplosimilarity score throughout the genome using sliding windows in order to detect haplotypes that contain alleles with low frequencies that are similar between each other, suggesting large haplotypes under a recent selective pressure that hasn’t been disrupted by recombination (Hanchard et al., 2006). |
|  | Integrated haplotype score (iHS) | The iHS compares the area under the curve defined by the EHH, which allows the identification of incomplete selective sweeps and soft sweeps throughout the genome (Voight et al., 2006). |
|  | Cross-population extended haplotype homozygosity (XP-EHH) statistic | This test compares the EHH in a locus between a population with a fixed haplotype against other populations where such locus remains polymorphic (*e.g.*, domesticated and wild populations), allowing it to detect selective sweeps after the selected allele reached fixation (Sabeti et al., 2007). |
|  | LD decay (LDD) test | The LDD test sorts individuals according to their homozygosity for each of the alleles found in any given SNP in the genome, and then calculates the fraction of heterozygous SNPs that are adjacent to each of the allelic variants in the SNP that is being evaluated. This way the LDD test can determine whether or not the LD decays significantly slower in one of the alleles of the evaluated SNP, suggesting a recent selective sweep. Since the test calculates the decay in LD only for the individuals that are homozygous in the SNP being evaluated, there is no necessity to obtain phased haplotypes (Wang et al., 2006). |
|  | Regression-based test | Calculates the reduction of heterozygosity as one approaches the locus under selection in a genome to infer selective sweeps (Wiener and Pong-Wong, 2011). |
|  | OmegaPlus | Implements the ω statistic by detecting regions with high SNP correlation coefficient across the genome to find regions under selection (Alachiotis et al., 2012). |
|  | GIBDLD | Calculates the identity-by-descent (IBD) between all pairs of individuals for each locus in the dataset to detect segments of IBD (*i.e.*, haplotypes) that are shared between several unrelated pairs of individuals, suggesting the action of selective pressures (Han and Abney, 2013). |
| Composite tests | Cross-population composite likelihood ratio (XP-CLR) test | The XP-CLR test searches for genomic regions with extended allele differentiation between a population under selective pressures (*i.e.*, domesticated taxa) and a "control" population (*i.e.*, a close wild relative) in order to detect the haplotypes where differentiation happened quicker than expected under neutrality (Chen et al., 2010). |
|  | RAiSD | This program uses the µ statistic to score genomic regions as candidate loci based on the joint analysis of LD, changes in the SFS and a reduction in the genetic diversity within sliding windows (Alachiotis and Pavlidis, 2018). |
